# Immunotherapy Deriving from CAR-T Cell Treatment in Autoimmune Diseases

**DOI:** 10.1155/2019/5727516

**Published:** 2019-12-31

**Authors:** Yuehong Chen, Jianhong Sun, Huan Liu, Geng Yin, Qibing Xie

**Affiliations:** Department of Rheumatology and Immunology, West China Hospital, Sichuan University, 37 Guoxue Xiang, Chengdu, Sichuan 610041, China

## Abstract

Chimeric antigen receptor T (CAR-T) cells are T cells engineered to express specific synthetic antigen receptors that can recognize antigens expressed by tumor cells, which after the binding of these antigens to the receptors are eliminated, and have been adopted to treat several kinds of malignancies. Autoimmune diseases (AIDs), a class of chronic disease conditions, can be broadly separated into autoantibody-mediated and T cell-mediated diseases. Treatments for AIDs are focused on restoring immune tolerance. However, current treatments have little effect on immune tolerance inverse; even the molecular target biologics like anti-TNF*α* inhibitors can only mildly restore immune balance. By using the idea of CAR-T cell treatment in tumors, CAR-T cell-derived immunotherapies, chimeric autoantibody receptor T (CAAR-T) cells, and CAR regulatory T (CAR-T) cells bring new hope of treatment choice for AIDs.

## 1. Introduction of Autoimmune Diseases

Autoimmune diseases (AIDs) are a spectrum of chronic disease conditions originating from an abnormal-activated autoimmune system and involve certain organ (organ-specific AIDs, i.e., type I diabetes, T1D) or multiple organ systems (systematic AIDs, e.g., systemic lupus erythematosus, SLE), displaying as autoimmune intolerance and leading to tissue injury [1–3]. Broadly, AIDs can be separated into two categories according to pathogenic mechanism: self-reactive antibody- or “autoantibody-” mediated AIDs in which antibodies are produced by plasma cells from the B lymphocyte lineage and self-reactive T lymphocyte-mediated AIDs. The incidence of AIDs is 80 cases per 100000 people, and the prevalence is over 3% globally, while in the USA, the prevalence reaches to 5%-8% [4, 5]. Women accounting for 65% of all patients, AIDs mainly occur in young and middle-aged women and have been the primary cause of death in the affected women. Currently, nearly a hundred kinds of AIDs have been reported, and the most common ones are T1D and autoimmune thyroid disease, followed by rheumatoid arthritis (RA), inflammatory bowel disease, SLE, and multiple sclerosis (MS) [6]. The definite etiologies of AIDs are unclear but may have association with genetic predisposition containing both monogenic and multiple genetic factors and environmental factors like nutrition, hormone level, diet, pathogens, drugs, insufficiency of vitamin D, and toxins [2, 7–9].

The pathogenesis of AIDs is not clear, but according to current study, the breakage of immune tolerance demonstrated when B or T lymphocytes fail to distinguish self from nonself with involvement of autoantibodies and/or self-reactive T lymphocytes is related to AIDs [2, 10]. The explanatory mechanisms to autoreactive B or T cells can be proposed as “molecular mimicry,” the most common mechanism, which is when the sequence of pathogen-derived peptides is similar with self-peptides, which causes cross-reactivity of antigen receptors and results in autoimmune response; “epitope spreading,” caused by virus infection, which is the change from the primary epitope to other epitopes or the generation of multiple neoepitopes on antigen-presenting cells; “bystander activation” which means the activation of preexisting autoreactive immune cells; and “viral persistence and polyclonal activation,” explained by continuous existence of viral antigen prompting immune response or epitope spreading. Moreover, other factors involved in regulating innate and adaptive immunity, like autoantigens released by apoptosis, microbiota, and insufficient vitamin D, may also contribute to loss of tolerance. All these mechanisms finally progress to reactive B or T cells and cause loss of immune tolerance and organ-specific or systemic autoimmune diseases [2, 3].

Autoantibody-mediated tissue destruction is a common feature of AIDs, which can be used to diagnose and classify AIDs [11]. Autoantibodies play a pathogenic role in cytotoxic damage by attacking a cell's functional structures through cell surface binding and lysis, and during the process, the most common damage pathways are complement activation and antibody-dependent cell-mediated cytotoxicity [2, 12]. SLE, Sjogren's syndrome (SS), and autoimmune hepatitis (AIH) are examples of autoantibody-mediated AIDs. Antigen-antibody immune complex-mediated tissue damage is also a critical pathogenic mechanism, and AIDs of SLE, RA, and SS are the illustrations. In addition, the selective pathways can be activated or blocked by autoantibodies after binding to cell surface receptors, and the activated selective disease Graves' disease and blocked selective disease myasthenia gravis are the instances. Self-reactive T lymphocyte-mediated AIDs are caused by cytotoxic effects. After recognizing a target cell by matching the T cell receptor (TCR) to the major histocompatibility complex I (MHCI) and autoantigen-originated peptides, autoreactive cytotoxic T cells directly kill target cells by secreting cytotoxic granules, like perforin and granzyme B, or activating the Fas-Fas ligand to induce cell apoptosis, and release cytokines like anti-tumor necrosis factor alpha (TNF*α*) and interferon gamma (IFN-*γ*) to cause tissue injury [2, 12, 13].

The key to treat AIDs is to restore immune tolerance. Traditionally, the typical immune suppressors are the disease-modifying antirheumatic drugs (DMARDs), like methotrexate, mycophenolate mofetil, cyclosporine, and so on, which give a general suppression of the immune system, thus causing increased risks of serious infection, developing lymphoma and other malignancies, but cannot significantly inverse immune tolerance. Recently, new immunosuppressants called biologics targeting localized targets or pathways rather than the whole immune system have been developed, like belimumab and rituximab depleting B cells, abatacept suppressing T cell activation, anti-TNF*α* inhibitors targeting TNF*α*, tocilizumab blocking interleukin 6 (IL-6), and ustekinumab inhibiting IL-12. The biologic agents are a class of monoclonal antibodies or fusion proteins targeting the receptors expressed by B cells or T cells or the key cytokines that involve regulation of B or T cells' differentiation. There are several ways to treat AIDs by targeting B lymphocytes, like eliminating B cells which is the direct method to wipe out the production of pathogenic antibodies, impeding B cells' activation by binding the inhibitory receptors expressed by B cells, or neutralizing key cytokines that participate in B cell activation, differentiation, or maturation [14]. Nevertheless, B cell elimination is the most widely used strategy to treat a series of AIDs, like RA, SLE, MS, and vasculitis.

Biologics can lower the toxicity and side effects in contrast with DMARDs, being better for long-term treatment. Nevertheless, they cannot restore the immune tolerance permanently [2, 15], thus requiring continuous administration, which brings new challenges, like weakened immunization of humanized antibodies [10, 15, 16]. Therefore, a precisely targeted treatment strategy that can restore immune tolerance is urgently needed. Fortunately, with the advances in adoptive cellular therapy for cancer, the extended use reaches AIDs.

## 2. Introduction of CAR-T Cells

The concept of adoptive cellular therapy (ACT) was first introduced when T cells were administrated to treat tumors, which benefited from the ability of IL-2 to grow human T cells ex vivo, therefore leading to the production of tumor-specific cells in a large scale. ACT has the advantages of expanding antitumor T cells ex vivo, high selection affinity towards target antigen, and modulating the host tumor microenvironment to a relative optimum condition prior to receiving T cell treatment. Later on, further use of ACT made the T cells have the engineered specific antitumor specificity by introducing conventional *αβ* TCR or synthetic constructs, chimeric antigen receptors (CARs), to recognize the antigen expressed by a tumor cell [17]. The structure of a TCR is more complex than a CAR. A TCR is composed of an *αβ* heterodimer which binds to peptide MHC, CD3 subunits, and a coreceptor CD4 or CD8 while a CAR consists of a single-chain molecule containing a single-chain variable fragment (scFv), a hinge, intracellular signaling domains from CD3*ζ*, and a costimulatory molecule [18–20]. So according to the structure, TCRs have lower affinities for their ligands than CARs, and antigen recognition of TCRs is dependent on MHC [21], while antigen recognition of CARs is not restricted by MHC which allows CAR-T cell treatment to have wider use by targeting more antigens, like proteins, carbohydrates, or glycolipids [19, 22, 23]. Therefore, more attention has concentrated on synthetic receptor CARs.

**Figure 1:**
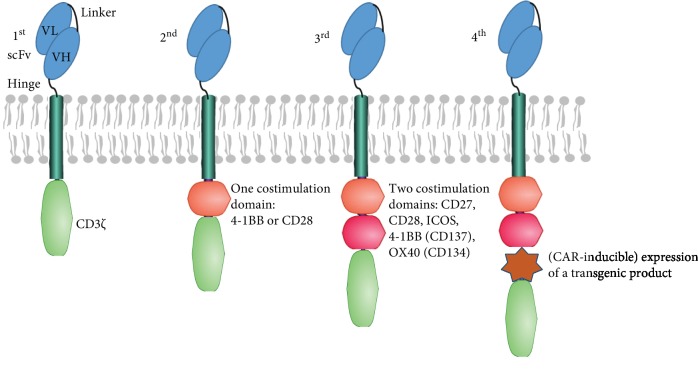
Generations of chimeric antigen receptors (CARs). CARs typically have an extracellular antigen recognition domain represented by an antibody-derived single-chain variable fragment (scFv) which contains a variable heavy (VH) chain and a variable light (VL) chain connected by a linker, a hinge, a transmembrane domain, with or without one or two costimulatory domains, and a CD3*ζ*. The fourth-generation CARs additionally have a “nuclear factor of activated T cell-responsive expression” element for an inducible transgenic product.

Chimeric antigen receptor T (CAR-T) cells are T cells engineered to express specific CARs that can recognize antigens expressed by tumor cells, and after the binding of antigens to receptors, the tumor cells are eliminated. The CAR-T cells can proliferate and survive in vivo for several years, which is the prerequisite for the treatment effect that keeps remission and controls or delays the relapse or deterioration of diseases [24]. To improve the antitumor efficacy and reduce T cell activation-accompanied toxicity of CAR-T cells, CARs went through an update, and the disparity for the different generations was presented in the intracellular signaling domain, the costimulatory domain. The first-generation CARs only have the CD3*ζ* intracellular domain, the second generations have both CD3*ζ* and one of the two costimulatory domains CD28 or 4-1BB (CD137), and third generations have two of the costimulatory domains such as CD27, CD28, ICOS, 4-1BB (CD137), or OX40 (CD134) in addition to CD3*ζ* [25, 26]. Compared to the third generation, the fourth-generation CAR-T cells, also called TRUCK T cells, are the CAR-T cells having a transgenic “payload” that is a “nuclear factor of activated T cell-responsive expression” element for an inducible transgenic product [27, 28] (Figure 1).

The application of CAR-T cells to treat B-lineage surface antigen CD19 is a huge forward step in cancer immunotherapy. The classic clinical use of CAR-T cells was to treat relapsed or refractory B cell acute lymphocyte leukemia (ALL), refractory B cell lymphoma, and non-Hodgkin lymphoma [29–32]. And now, the CAR-T cell is designed to have wider use that is to treat B cell malignancies beyond ALL, like chronic lymphocytic leukemia and multiple myeloma [24, 33, 34]. Also, CAR-T cells were used to target other hematological B cell, T cell, or myeloid malignancies; antigen molecular targets like BCMA, CD20, CD30, CD33, CD70, and CD123; and solid cancers like renal cell carcinoma (targeting CAIX), neuroblastoma (targeting L1-CAM or GD2), colon adenocarcinoma (targeting ErbB2), mesothelioma and pancreatic adenocarcinoma (targeting mesothelin) [17], sarcoma, antigen-targeted molecules like B7H3 [22, 25, 35, 36], and relapsed or refractory B cell precursor ALL [37].

Although CAR-T cell treatment has achieved huge success in hematologic malignancies, reaching high remission rate and response rate, treatment-related toxicity should be a concern, which is caused by high levels of inflammatory cytokines during the T cell activation and proliferation. Among the CAR-T cell treatment-related adverse events, cytokine release syndrome (CRS) is the most common one [38–41]; other adverse events like neutropenia, anemia, thrombocytopenia, and neurologic events are also common [29, 30].

## 3. CAR-T Cell-Derived Immunotherapy in AIDs

Application of CAR-T cells in AIDs to reach target treatment is promising [16, 42–44].

Similar to tumor treatment by targeting tumor-associated antigens expressed on the surface of tumor cells, CAR-T cells can be modified to treat AIDs by targeting specific autoantigens or antibodies expressed on the pathogenic cell surface. CAR-T cell-derived immunotherapy for AIDs can be classified as treatments of chimeric autoantibody receptor T (CAAR-T) cell and CAR-Treg based on a pathogenic mechanism. Several preclinical studies have been performed to investigate the application of CAR-T cells in AIDs (Table 1), and a few clinical trials are ongoing (Table 2).

CAAR-T cells are modified from CAR-T cells where chimeric autoantibody receptors are harbored by T cells instead of chimeric antigen receptors to target cells secreting antibodies, the autoreactive B cells [15], so the construction of a CAAR-T cell is composed of a specific antigen, a transmembrane domain, and intracellular signaling domains. After the specific antigen of the CAAR-T cells recognize and bind to the cognate autoantibodies expressed by the specific antibody producing B cells, the B cells will be eliminated. Using CAAR-T cells to treat antibody-mediated AIDs, two preconditions are needed [15]. One is that the sequence and molecular structure of the specific antigens are clear to guarantee the engineered key epitopes of the CAAR are correct to make sure the engineered epitopes can be recognized by cognate autoantibodies from patients. The other is the role of autoantibodies in the pathogenesis of a disease should be well investigated to make sure their pathogenicity.

Dsg3 CAAR-T cells were the human T cells engineered to express a CAAR that consisted of the pemphigus vulgaris (PV) autoantigen and desmoglein 3 (Dsg3), fused to CD137-CD3*ζ* signaling domains, and were effective for PV relief without any off-target toxicity, specifically eliminating Dsg3-specific B cells, thus obviously decreasing Dsg3 serum autoantibody titers [42]. Using Dsg3 CAAR-T cells to treat PV is representative for applying CAR-T cells targeting antibody-mediated AIDs. Nevertheless, owing to short-term observations, the safety and efficacy are yet to be confirmed.

In murine lupus, CD8+ T cells were modified to express CD19-targeted CARs with CD28-CD3*ζ* signaling domains. A single use of CD19-targeted CAR-T cells was highly effective to treat lupus, manifested as complete and sustained CD19+ B cell depletion, terminated autoantibody production, reversed disease phenotype, and prolonged survival time, and the treatment effect was sustained for up to one year. Transferring splenic T cells from the mice after CD19+ B cell depletion by CAR-T cell treatment to lupus prone mice alleviated disease severity in adoptive autoimmune mice [45]. The persistence and function of CD19-targeted CAR-T cells were quite long in vivo after a single administration, reaching up to one year, and meanwhile, persistent B cell depletion was observed. This CD8+ T cell-originated CD19-targeted CAR-T cells targeted cell death in a direct mechanism way that depleted B cells effectively without the help from other cell types, which is superior to antibody-mediated cytotoxicity that requires a binding antibody for complement-dependent cell lysis. For instance, treatment of rituximab, an anti-CD20 antibody, requires repeated use to reach a therapeutic dose, with an insufficient required dose resulting in incomplete B cell depletion and failure treatment. Also, in this study, no obvious side effects were observed with the treatment of CD19-targeted CAR-T cells. But studies are required to further figure out the trait and function of plasma cell population and residual IgM^lo^ B cell in the murine lupus model. Therefore, CD19-targeted CAR-T cell treatment seems to be a new hope for SLE patients by targeting depletion antibody-producing B cells [46–48].

In the T1D NOD mouse model, I-A^g7^-B:9-23 (R3) refers to a pathogenic complex wherein the MHC class II molecule I-A^g7^in register 3 (R3) binds to the B:9-23 peptide which is a primary initiating epitope located between residues 9 and 23 of the insulin B chain, and a monoclonal antibody named mAb287 that can selectively bind to this complex was generated. 287-CAR-T cells were CD8+ T cells modified to target the I-A^g7^-B:9-23 (R3) complexes, which could only delay the onset of T1D for about 6 weeks with a single infusion but could not prevent the disease development owing to short-time persistence of the transferred cells, having no detectable cells at 25 weeks of age [49]. This is the first study to demonstrate that CAR-T cells can be used to selectively target pathogenic T cell epitopes associated with autoimmunity presented by antigen presentation cells (APCs). The 287-CAR-T cells seemed unable to proliferate or survive in the spleen; however, they could home in on and expand in pancreatic lymph nodes where they possessed their cognate antigens as expressed by APCs. Also, those adoptively transferred 287-CAR-T cells could migrate to the target tissue, which are the inflamed islets. Application of 287-CAR-T cells did not show any adverse metabolic side effects and the gross change of immune cell populations and percentages.

Regulatory T cells (Tregs) also play a critical role in regulating the immune system by inhibiting the function of immune cells to keep immunologic self-tolerance and immune homeostasis, and an AID will occur when the specific transcription factor Forkhead box protein P3 (Foxp3) of Tregs is mutated or the CD4+CD25+ T cells are eliminated [50]. Therefore, applying Treg therapy in AIDs after being engineered to CAR-Tregs having antigen specificity may be a new choice [16, 21, 51, 52]. CAR-Tregs can induce antigen-specific cytolysis of the targeted cell in a granzyme B-dependent way, suppressing antigen-specific effector T cells' (Teffs) response, and releasing immunosuppressive cytokines, like transforming growth factor *β*1 (TGF-*β*1) and IL-10 [53]. CAR-Tregs are transduced from T cells and expanded ex vivo with normal expressing levels of Foxp3 to keep the expanding ability of reaching the therapeutic number, but transformation from CAR-Tregs to effector CAR-T cells in an inflammatory milieu is the major safety issue [44]. Nevertheless, CAR-Tregs can suppress Teffs by the following mechanisms: releasing immunosuppressive cytokines, such as IL-10, IL-35, and TGF-*β*; competing binding molecules CD80/CD86 on APCs with cytotoxic T lymphocyte antigen 4 expressed by CAR-Tregs to CD28 expressed by Teffs; and inducing apoptosis of Teffs through Fas-ligand or granzyme B/A and perforin produced by CAT-Tregs [52].

Insulin-specific CAR converted Tregs (CAR-cTregs), the first CAR-Tregs for T1D, are engineered CAR-Tregs where CD4+ T cells were transduced with retroviral particles encoding the second-generation CAR plasmid including Foxp3 to convert CD4+ T cells into Tregs. In the presence of insulin, proliferation of CAR-cTregs in vitro was normal and the suppressive capacity was similar to natural Tregs [54]. Although CAR-cTregs have a long existence in diabetic mice that they could be detected as long as at the 17th week after the adoptive transfer, they could not prevent spontaneous diabetes in NOD/Ltj female mice. The possible explanation for prevention failure of diabetes is that the soluble hexamer rather than the soluble monomer can activate CAR-T cells. As the specificity of CAR-cTregs is high with the antigen insulin, off-target effects are supposed to be small [54].

The engineered MOG CAR-Tregs are CD4+ T cells transduced to Tregs expressing CARs with myelin oligodendrocyte glycoprotein (MOG), the FoxP3 gene, and CD28-CD3*ζ* signaling domains. MOG CAR-Tregs were effective to treat autoimmune encephalomyelitis (EAE), a model that mimics multiple sclerosis in humans after a single intranasal delivery and which homes in on various regions in the brain. The treatment reduced disease symptoms and decreased mRNA expressions of cytokines IFN-*γ* and IL-12. The EAE scores were instantly decreased upon intranasal administration, and the reduction of clinical disease symptoms was continuous, even becoming symptom-free on the 25th day. Intranasal delivery of CAR-Tregs was addressed when treating EAE as a cell numbers which would be decreased when homing in on the target tissue. So transplantation of cells into the brain through intranasal delivery can reduce cell dose and systemic exposure [55].

2,4,6-Trinitrophenol (TNP) tripartite chimeric receptor (TPCR) natural Tregs (TNP-TPCR Tregs) are natural Tregs isolated from transgenic mice expressing the TNP-specific chimeric receptor under the CD2 promoter with a maintained high Foxp3 level. TNP-TPCR Tregs could repeatedly expand when stimulated by cognate antigen ex vivo in a costimulation-independent and contact-relying way and were effective to alleviate acute 2,4,6-trinitrobenzenesulphonic acid- (TNBS-) induced colitis in a dose-dependent manner. After recovery from the first TNBS stimulation for three weeks, the second time TNBS colitis was inducted in TNP-TPCR Treg-treated mice showed 75% survival of mice, higher than the 33% survival in the wild-type Treg-treated group, indicating the development of persistent tolerance. After activation by dendritic cells preloaded with TNP, TNP-TPCR Tregs could inhibit proliferation of Teffs in a dose-dependent way [56].

Carcinoembryonic antigen- (CEA-) CAR-Tregs were the CD4+CD25+ Tregs transduced with the CEA-specific SCA431 CAR that was fused to CD28-CD3*ζ* signaling domains, and about 90% of the CAR-Tregs were FoxP3-positive cells. CEA-CAR-Tregs were effective for T cell transferred colitis relief and in inhibiting the development of the azoxymethane-dextran sodium sulfate- (AOM-DSS-) induced colitis-associated colorectal cancer [57]. CEA-CAR-Tregs could be homed in on and accumulated to the CEA-expressed sites, with the highest detection in the inflamed colon and to a much lesser extent in the small intestines and no detection in other visceral organs. The persistence of the CEA-CAR-Tregs was short, only accumulating and expanding in the colon for roughly 7 days and then quickly fading away in that it could not be detected at the 9th day after injection [57].

The clinical trial of NCT04146051 is a single-group nonrandomized study planning to enroll 18 participants to assess the safety and preliminary efficacy of CAR-T cells engineered from autologous T cells containing descartes-08 drug targeting B cell maturation antigen in patients with generalized myasthenia gravis. The trial has two phases, Ib and IIa. Phase Ib is a dose-escalation phase to measure the outcomes of the maximum tolerated dose with follow-up time for 28 days, and phase IIa is an expansion phase to observe the change of the daily living score during the 168-day follow-up period.

The clinical trial of NCT03030976 is a single-arm open-labeled nonrandomized study to assess the safety and efficacy of CD19-CAR-T cells engineered from autologous T cells with a second CAR containing 4-1BB as a costimulator in patients with CD19 positive B cell SLE. The trial is a phase I study intending to enroll 5 patients, and two days ahead of an initial infusion of (1-10) E6 CAR-positive T cells/kg, cyclophosphamide (0.5 g/m^2^/d) is applied to reduce B cells. Assessment of safety is to report the number of adverse events, and efficacy is the overall response rates and the persistence of infused CAR-T cells in the circulation detected by quantitative PCR during the 6-week follow-up period.

## 4. Future Prospects

With advances in updating of constructing CARs and accumulating preclinical studies, application of CAR-T cell-derived immunotherapy in AIDs is feasible. Although preclinical studies have been performed, there is still a long way before we can apply CAR-T cells to clinical treatment in AIDs. Before clinical use, safety, effectiveness, persistence, and manufacture of CAR-T cells must be guaranteed. Using CAR-T cells to treat AIDs, CARs can be tailored according to specific antigens or antibodies in different AIDs, so CAR-T cells have unique specificity. Therefore, treatment of CAR-T cells theoretically does not cause side effects. Nevertheless, finding the specific antigens to construct antigen-specific CARs is not easy in some disease conditions. As CAR-T cells recognize cell surface molecules without the help of human leukocyte antigen expression, antigen recognition of CARs is not restricted by MHC, and CAR-T cell may recognize almost all types of antigens like carbohydrates, lipids, and proteins [27]. Expansion CAR-T cells in a large scale for clinical use may be challengeable, which can be solved by a cell culture platform [58, 59].

Exhaustion of CAR-T cells limits their functions in immunoregulation. As the costimulatory domains play a key regulatory role in determining functionality and persistence of CAR-T cells both in vitro and in vivo, costimulatory domains are the targets to improve the persistence. Although CARs with CD28 are related to enhanced expansion, persistence, and antitumor effect [60, 61], CARs with CD28 are not as good as CARs with 4-1BB (CD137) [62–64]. The persistence and antitumor effect of CARs with CD27 are similar to the CARs bearing CD28 or CD137 [65]. CD28-OX40 CARs can enhance specific cytolysis and improve antitumor response [66]. CARs with ICOS can further increase persistence and antitumor response in contrast with CD28- or CD137-alone CARs [67]. However, the suitable combinations of those costimulatory domains to reach the best persistence need more studies. In addition to modulating costimulatory domains in the first three-generation CARs to improve the persistence and expansion of CAR-T cells in vivo, the fourth-generation CARs are promising [28], as they have an inducible expression cassette for a transgenic protein, so those factors of IL-2 receptor *β*-chain, mRNA-encoding telomerase reverse transcriptase, or PI3K inhibitor should be considered when constructing CARs [68–70].

For the use of CAR-Tregs, attention should be paid to several issues. On the on hand, the immunosuppressive phenotype of Tregs will change after losing Foxp3 expression under an inflammatory microenvironment, from the immunosuppressive state to effector cells that aggravate disease symptoms [10]. To maintain the immune inhibitory phenotype of Tregs, several methods can be tried, like treating the Tregs with the vitamin A derivative all-trans retinoic acid that can sustain the stability and functionality of Tregs [71], administrating a Treg-favoring microbiota to the gut [72], and inducing ectopic expression of the Foxp3 gene to a stable regulatory phenotype of Tregs [73]. On the other hand, CD28 is the most critical costimulatory pathway to keep Tregs homeostasis plays a critical role in Tregs proliferation, differentiation, and survival and can upregulate IL-2 production and Foxp3 expression [74]. So CD28 should be adopted in CARs when constructing CAR-Tregs.

## Figures and Tables

**Table 1:** CAR-T cell-derived immunotherapy in preclinical models of AIDs.

CARs/generations	Construction of specific T cells or Tregs	Disease model	Specific autoantigen	Outcome	Reference
Dsg3 CAAR-T cells/2nd	Human T cells were engineered to express a CAAR that consisted of the PV autoantigen, Dsg3, fused to CD137-CD3*ζ* signaling domains	PV	Dsg3	Effective for PV relief, obviously decreased Dsg3 serum autoantibody titers	Ellebrecht et al. [42]
CD19-targeted CAR-T cells/2nd	CD8+ T cells were modified to express CD19-targeted CARs with CD28-CD3*ζ* signaling domains	Lupus	CD19	Eliminated autoantibody production, reversed disease phenotype, and prolonged survival time	Kansal and Richardson [45]
287-CAR-T cells/2nd or 3rd	CD8+ T cells were modified to express monoclonal antibody 287 CARs (287-CARs) harboring CD28-CD3*ζ* or CD28-CD137-CD3*ζ* signaling domains	T1D	I-A^g7^-B:9-23 (R3) complex	Delayed but did not prevent the onset of T1D	Zhang et al. [49]
Insulin-specific CAR-Tregs/2nd	CD4+ T cells were transduced with retroviral particles encoding the CAR plasmid including Foxp3 to convert CD4+ T cells into Tregs	T1D	Insulin	Failed to prevent spontaneous diabetes	Tenspolde et al. [54]
MOG CAR-Tregs/2nd	CD4+ T cells transduced to Tregs expressing CARs with MOG, the FoxP3 gene, and CD28-CD3*ζ* signaling domains	MS	MOG	Inhibited EAE demonstrated as reduced cytokine expression and diminished disease symptoms	Fransson et al. [55]
TNP-TPCR Tregs	TNP-TPCR Tregs were isolated from transgenic mice expressing the TNP-specific chimeric receptor under the CD2 promoter	Colitis	TNP	Alleviated acute TNBS colitis	Elinav et al. [56]
CEA-specific CAR-Tregs/2nd	CD4+CD25+ Tregs were transduced with the CEA-specific SCA431 CAR that was fused to CD28-CD3*ζ* signaling domains	Colitis and colorectal cancer	CEA	Ameliorated colitis and prevented development of colitis-associated colorectal cancer	Blat et al. [57]

PV: pemphigus vulgaris; T1D: type I diabetes; Dsg3: desmoglein 3; MS: multiple sclerosis; MOG: myelin oligodendrocyte glycoprotein; EAE: encephalomyelitis; TPCR: tripartite chimeric receptor; TNP: 2,4,6-trinitrophenol; TNBS: 2,4,6-trinitrobenzenesulphonic acid; CEA: carcinoembryonic antigen.

**Table 2:** Clinical trials of CAR-T cell treatment for AIDs.

Intervention	Disease condition	Phase	Status	ClinicalTrials.gov identifier	Institute
Descartes-08 CAR-T cells	Generalized myasthenia gravis	I and II	Recruiting	NCT04146051	University of Miami
Anti-CD19 CAR-T cells	Systemic lupus erythematosus	I	Recruiting	NCT03030976	Shanghai Jiaotong University School of Medicine, Renji Hospital
